# Assessing the Antimicrobial Activity of Polyisoprene Based Surfaces

**DOI:** 10.3390/ijms16034392

**Published:** 2015-02-19

**Authors:** Hope Badawy, Jérôme Brunellière, Marina Veryaskina, Guillaume Brotons, Sophie Sablé, Isabelle Lanneluc, Kelly Lambert, Pascal Marmey, Amy Milsted, Teresa Cutright, Arnaud Nourry, Jean-Luc Mouget, Pamela Pasetto

**Affiliations:** 1 LUNAM Université, Institut des Molécules et des Matériaux du Mans, UMR CNRS 6283, Université du Maine, Avenue Olivier Messiaen, 72085 Le Mans, France; E-Mails: hope.badawy@gmail.com (H.B.); mveryaskina@mail.ru (M.V.); guillaume.brotons@univ-lemans.fr (G.B.); arnaud.nourry@univ-lemans.fr (A.N.); 2 Centre de Transfert de Technologie du Mans, 20 rue Thalès de Milet, 72000 Le Mans, France; E-Mails: jbrunelliere@cttm-lemans.com (J.B.); klambert@cttm-lemans.com (K.L.); pmarmey@cttm-lemans.com (P.M.); 3 LIENSs, UMR CNRS 7266, Université de La Rochelle, Bât. Marie Curie, Av. Michel Crépeau, 17042 La Rochelle, France; E-Mails: ssable@univ-lr.fr (S.S.); isabelle.lanneluc@univ-lr.fr (I.L.); 4 Biology Department, University of Akron, Akron, OH 44325, USA; E-Mail: milsted@uakron.edu; 5 Department of Civil Engineering, University of Akron, Akron, OH 44325, USA; E-Mail: tcutrig@uakron.edu; 6 MMS-Mer, Molécules-Santé, FR CNRS 3473 IUML, Université du Maine, 72085 Le Mans, France

**Keywords:** natural rubber, antibacterial surfaces, environmentally friendly material, antifouling coating

## Abstract

There has been an intense research effort in the last decades in the field of biofouling prevention as it concerns many aspects of everyday life and causes problems to devices, the environment, and human health. Many different antifouling and antimicrobial materials have been developed to struggle against bacteria and other micro- and macro-organism attachment to different surfaces. However the “miracle solution” has still to be found. The research presented here concerns the synthesis of bio-based polymeric materials and the biological tests that showed their antifouling and, at the same time, antibacterial activity. The raw material used for the coating synthesis was natural rubber. The polyisoprene chains were fragmented to obtain oligomers, which had reactive chemical groups at their chain ends, therefore they could be modified to insert polymerizable and biocidal groups. Films were obtained by radical photopolymerization of the natural rubber derived oligomers and their structure was altered, in order to understand the mechanism of attachment inhibition and to increase the efficiency of the anti-biofouling action. The adhesion of three species of pathogenic bacteria and six strains of marine bacteria was studied. The coatings were able to inhibit bacterial attachment by contact, as it was verified that no detectable leaching of toxic molecules occurred.

## 1. Introduction

The development of surfaces that resist the adhesion and colonization of microbes is an ongoing industrial and academic research topic for applications concerning things of everyday usage, and it is of crucial importance in the biomedical environment. Many different approaches have been attempted to create antimicrobial materials [1,2,3], such as surface modification by plasma technique [4,5], synthesis of composites formed by a polymer and inorganic particles as biocidal agents [6], synthesis of polymers bearing antibacterial groups in their backbone or chain ends [7], and polymers containing a small biocide molecule that is released in appropriate conditions [8].

In an aquatic environment, biofouling can cause substantial economic losses for many industrial sectors (e.g., sensors, shipping [9], and aquaculture [10]), due to the development of microscopic and macroscopic organisms on submerged surfaces. A key step for the consolidation of these communities is the adsorption of macromolecules (e.g., proteins) and the successive formation of a biofilm of microorganisms, composed of an organic matrix (primarily polysaccharide material) with bacteria, fungi, and microalgae, mainly diatoms. The demand for environmentally-friendly systems to prevent the formation of biofilms and control the fouling on surfaces has urgently increased in the last decade, due to environmental constraints and new regulations (for recent reviews see Banerjee *et al.* [11], Gittens *et al.* [12]). Most traditional and now banned antifouling materials typically contained biocidic agents that were ultimately released in the environment. New approaches for the development of environmentally-friendly antifouling coatings [12] rely on the absence of any release of toxic agents in the environment [13].

In the polymer synthesis field and more widely in the industrial production of plastics, an emerging need/trend is to replace petroleum-derived monomers with the same or analogous ones derived from natural resources [14], in order to produce the same objects but starting from raw materials from renewable sources such as corn, potatoes, sugarcane, and trees [15]. Even if most brands are proud to present their bioplastic packaging to the customers, the latter are rarely aware that the legislation allows calling “bioplastic” a material that is only 20%–30% derived from a natural resource, while the remaining percentage comes from identical fossil-derived monomers. Moreover, the use of land to grow crops destined for industrial applications and not for alimentation has already led to aberrations, as in Brazil or Indonesia, where farmers have cut down rainforests to plant crops for the production of biodiesels, with the additional consequence that the prices of traditional crops have risen and not all the population can afford to pay more for food.

In the present work, the societal needs of having antibacterial/antifouling and biosourced materials have been combined by synthesizing bioactive films from natural rubber (NR) derived functional oligomers. In our laboratory a methodology has been optimized to cut the polyisoprene chains of natural rubber into telechelic oligomers that bear functional groups at the chain ends, which allows performing further chemical reactions. Natural rubber is not a crop used for food, therefore its use does not present the inconvenience mentioned before; its production is already stable and well established, as it is used for many mass produced goods.

In a previous work [16], various formulations were prepared to generate polymeric films using different NR oligomers bearing quaternary ammonium groups as bioactive functions as well as polymerizable groups. It emerged that the presence of oligomers containing one or three quaternary ammonium groups increased the antimicrobial activity in comparison to coatings that did not contain them. As a small but noticeable activity was observed even for NR-derived coatings that did not contain any ammonium but only the polyisoprene backbone alone, the study has been continued to understand the origin of this effect obtained without adding any compound or chemically modifying the structure, and to develop environmentally-friendly materials with high anti-fouling (AF) activity. This work has become the background for grafting groups that enhance the baseline activity of the NR-derived backbone, to design formulations composed of different types of NR-based oligomers, or to add a non-toxic molecule that could be slowly released in the medium. The anti-fouling ability of our coatings was tested using different strains of bacteria relevant to the two major fields of potential applications, pathogenic species for the biomedical environment, and marine benthic species for the microfouling and biocorrosion aspects. The different formulations of the NR-based coatings, tested in the biological assays using either pathogenic or marine bacteria, are presented here.

## 2. Results and Discussion

The results presented in this paper concern the study of the biological activity of polyisoprene based surfaces. The starting material used to prepare the films was obtained from natural rubber. After dissolution in tetrahydrofuran, NR was submitted to an oxidative treatment with periodic acid, at low temperature (30 °C) and atmospheric pressure, to cut some of the polyisoprene carbon-carbon double bonds and to obtain shorter oligoisoprenes possessing an aldehyde and a ketone at their chain ends [17]. The amount of periodic acid was added considering the number of moles of double bonds to cut statistically in order to have oligomers with a targeted molecular weight (two molecules of H_5_IO_6_ are required for one double bond). This procedure allowed the preparation of oligomers of different molecular weights in the range of 700–15,000 g·mol^−1^, with dispersities around 2. Successive reactions could be carried out to modify the extremities in order to insert different chemical groups. The strategy used for the antimicrobial materials was to insert one or two acrylate groups in the oligomer structure to perform radical polymerization and to have linear or cross-linked polymeric films, and to insert also a biocide functionality covalently linked, in order to obtain non-releasing antibacterial surfaces. The previous tests with both pathogenic and marine bacteria [16,18], showed that the presence of a single or of three quaternary ammonium groups (QA) per oligomer provides a surface that has both antifouling and antibacterial effect. However, as surfaces containing only the polyisoprene backbone, prepared from acrylate oligomer **(1)** without any biocide group, showed a low but noticeable activity, the present study focused on the synthesis of this kind of “simple” film and on the modification of a few features in order to have indications about the reasons of the intrinsic activity, which can be enhanced by grafting biocide groups.

For most of the biological tests, regarding the biomedical environment, the three pathogenic bacteria used previously [16] were chosen, one Gram-negative (*Pseudomonas aeruginosa*, PA), and two Gram-positive (*Staphylococcus aureus*, SA, and *Staphylococcus epidermidis*, SE). Concerning antifouling in the marine environment context, different wild bacteria strains were tested: four Gram-negative strains (*Flavobacterium* II2003, *Shewanella* IV3014, *Alteromonas* IVA014, and the pathogenic *Vibrio splendidus*), and two Gram-positive strains (*Bacillus* IV3004, *Bacillus* IVA016). They were selected for their ability to form biofilm and/or be involved in biocorrosion processes.

An attachment assay was performed for each strain and surface. The assay was looking at the initial stage of biofilm formation, where within a short period of time cells have already committed to attachment. A quantitative plate count was performed as opposed to simply looking at the optical density in order to determine living cell numbers and viability.

### 2.1. Effect of Acrylate and Ammonium Oligomer Molar Mass

**Figure 1:**
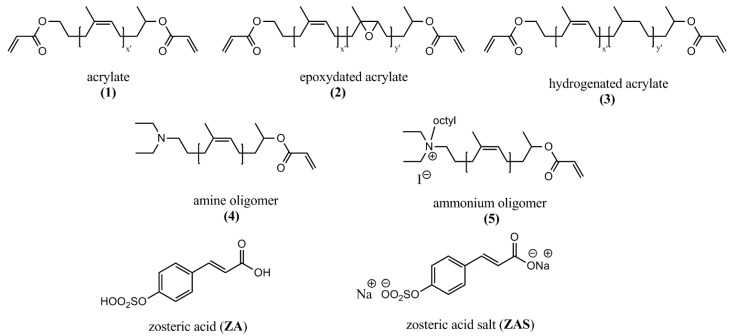
Structures of natural rubber derived oligomers and molecules used in this work.

The first biological studies were carried out on surfaces obtained from the acrylate oligomers **(1)** (Figure 1). The parameters used to perform the polymerization were optimized and described in a previous publication [17], therefore they will not be discussed in detail here. The oligomer **(1)** possesses two acrylate groups; therefore cross-linked films were formed by the photopolymerization of the liquid viscous mixture of acrylates and (5% weight) of photoinitiator, deposited on the flat bottom of a well of a 24-well cell culture plate (50–70 mg in each). This system proved to be the most suitable to insert the suspension of bacteria in contact with the polymers, having several replicates each time and a control polystyrene surface on the same plate. The “acrylate” thick films were yellowish but transparent, non-significantly swelling and flexible. The thermogravimetric analysis (TGA, the thermogram is shown in Figure S1) showed that they start to decompose after heating at 180 °C, which means they could be sterilized in an autoclave at 100 °C if needed for a specific application.

A procedure was optimized to wash the films after their synthesis and make sure that in the conditions of the biological assays, no leaching was occurring from the polymers. Plates were washed three times in sterile water and sonicated for eight seconds. No leaching being indeed a prerequisite for the possible development of environmentally-friendly polymers, an experiment to assess the toxicity of a medium having been in contact with the NR coatings was carried out. Surfaces obtained from the acrylate oligomers **(1)** (1700 g·mol^−1^) were incubated with nutrient broth for 3 h and 24 h, at 37 °C. Then the supernatant media were removed to a fresh plate not containing the polymers and two different bacterial strains, *S. aureus* (SA) and *Escherichia coli* (EC), were added and normal 3 h growth assays were carried out (Figures S2). No difference in the growth curves was observed between bacteria grown in a fresh medium and bacteria grown in supernatant media that had been in contact with the coatings for 3 h or 24 h. This indicated that no leaching of toxic molecules had occurred in the conditions adopted for the biological tests. To demonstrate that the test was sensitive enough to evidence possible leaching, we ran the same experiment using a surface made with ammonium oligomers **(5)** (1700 g·mol^−1^) but not completely cured, thus forming an incomplete three dimensional network from which small oligomers, unreacted photoinitiator, or impurities could be released. The same experiment described above was performed, with nutrient broth in contact with the polymer for 3 h or 24 h, and supernatants used as growth medium (3 h) for SA and EC. At the end of the 3 h growth period, the bacterial number decreased significantly, demonstrating that they had been negatively affected. To conclude, this means that if the curing process is effective and the film is completely formed, the resulting materials are non-leaching.

In a first series of experiments, two different batches of acrylate oligomers were synthesized; the first one with molecular weight (MW) of 1700 g·mol^−1^ and the second one with MW of 4000 g·mol^−1^, to see if the chain length had any effect on the antibacterial properties. The influence of the oligomer molecular weight on the photopolymerization process was reported in a previous paper [17], however the surfaces were not submitted to biological tests. It was shown that conversion was lower for longer oligomers (98% for MW of 2000 g·mol^−1^ *versus* 80% for MW 4000 g·mol^−1^) because their superior viscosity reduced the chains mobility and limited the diffusion of active centers, leaving statistically unreacted chain ends.

**Figure 2:**
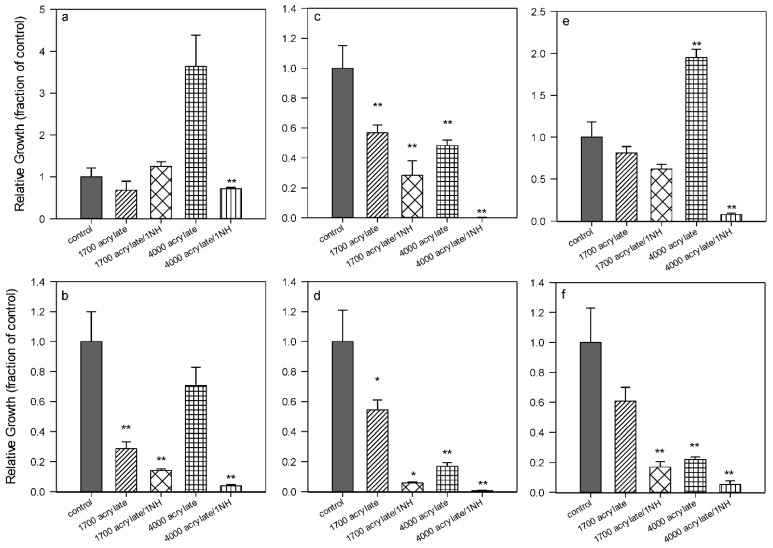
Effect of acrylate **(1)** and ammonium oligomer **(5)** chain lengths. *Pseudomonas aeruginosa* (**left**), *Staphylococcus aureus* (**middle**), *Staphylococcus epidermidis* (**right**). (**a**), (**c**), (**e**) = bacteria suspended in the supernatant medium; (**b**), (**d**), (**f**) = bacteria attached to the control (polystyrene) or polymer surface. *, difference in values significant at *p* ≤ 0.05, ** difference in values significant at *p* ≤ 0.01.

The films generated from both oligomers **(1)** revealed to have an activity in comparison to the control surface (polystyrene). For the three pathogenic species tested (PA, SE, SA), there were fewer cells on the acrylate-based surfaces than on the control (Figure 2). It seems that the chain length had no influence in the case of thick films, because in the case of bacteria SE and SA the shorter chain was more effective, but it was the contrary for PA.

In the suspension, the smaller oligomers were always effective in reducing the number of bacteria in comparison to the initial concentration, while the oligomers with higher molecular weight were not.

The oligomers **(5)** bearing one ammonium group (Figure 1) were also synthesized with the two different molecular weights and, as they have only one acrylate bond, the relative films were formed by linear polymers. As already observed, the presence of the ammonium group in the structure increased in every case the activity of the polymer surface in comparison to the acrylate surfaces alone and to the control; the same effect was noticed in the suspension (Figure 2). The longer ammonium oligomers produced also the most active surfaces in comparison to the shorter ones and this effect was observed as well in the suspension medium. A similar effect of a higher microbiocidal activity from longer polymer chains was found by Lin *et al.* [19]. They covalently grafted *N*-hexylated, *N*-methylated-polyethyleneimine (PEI) chains to amino functionalized glass slides and incubated them with airborne *Staphylococcus aureus*. It was found that chains of MW 750 and 25 kD were almost completely lethal to the microbes, while the 2 and 0.8 kD ones had a negligible activity, similar to the grafted analogous monomer diaminoethane. The authors tried to estimate the PEI chains lengths and compared them to the dimensions of the bacteria; they found that the 750 and 25 kD chains could be able to completely or partially penetrate into the cell wall and cell membrane and disrupt it, provoking bacterial death. On the other hand, the 2 and 0.8 kD chains were not long enough to considerably damage the cell structure. It is possible that an analogous mechanism takes place in the case of the ammonium groups of the oligoisoprenes, which could explain their antimicrobial effect. However they have a linear structure while the PEI used by Lin *et al.* [19] had a branched structure, with QA all along the polymer backbone.

The results pointed out that in future work the study of the influence of the oligoisoprene chain length should be pursued, whatever biocide group is attached, in order to assess the optimal molecular weight to use to enhance the antibacterial activity. It was already demonstrated [16] that formulations can be composed by a mixture of acrylate oligomers **(1)** and ammonium oligomers in different percentages, and that an active surface can be obtained using a minimum amount of ammonium oligomers. The new indication is that, in future formulations, the MW of the acrylate oligomers has no influence but the global activity can be improved playing with the parameter of the chain length of the oligomer containing the biocide.

### 2.2. Influence of Partial Epoxidation or Hydrogenation of Acrylate Backbone

**Figure 3:**
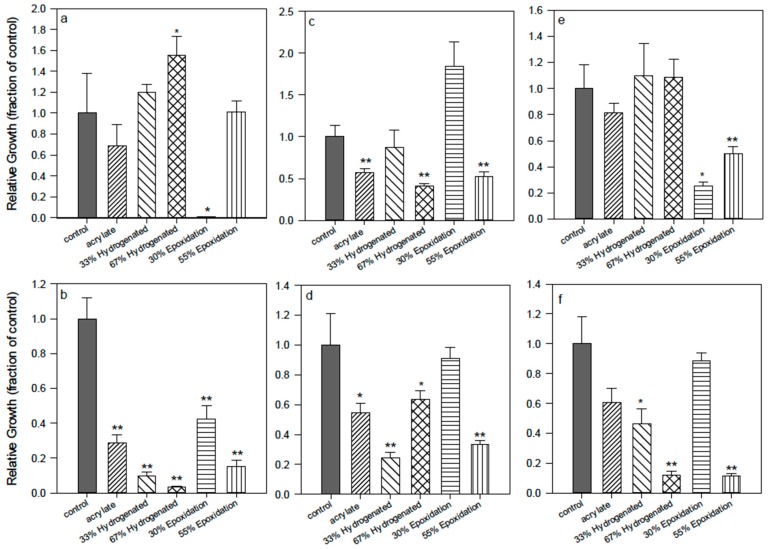
Effect of hydrogenation or epoxidation of acrylate **(1)**. 33%–67% hydrogenation = oligomer **(3)**; 30%–55% epoxidation = oligomer **(2)**. *Pseudomonas aeruginosa* (**left**), *Staphylococcus aureus* (**middle**), *Staphylococcus epidermidis* (**right**). (**a**), (**c**), (**e**) = bacteria suspended in the supernatant media; (**b**), (**d**), (**f**) = bacteria attached to the control (polystyrene) or polymer surface. *, difference in values significant at *p* ≤ 0.05, ** difference in values significant at *p* ≤ 0.01.

In order to have insights about the reasons of the basic attachment inhibition exerted by the polyisoprene surfaces, it was decided to focus the attention on the polymer backbone constituted by the sequence of *cis* carbon–carbon double bonds of the isoprene units, and to see if the suppression of some of them by hydrogenation or epoxidation could cause any change. Two different versions of the acrylate oligomers were synthesized: the epoxydated acrylates **(2)** (Figure 3), in which 30% or 55% of the double bonds were oxidized (transformed into epoxy groups), and the hydrogenated acrylates **(3)** (Figure 3), in which 33% or 67% of the double bonds were reduced by catalytic hydrogenation (see also Scheme S1). Cross-linked films were obtained in conditions similar to those described here in Section 2.1 through radical photopolymerization and tested with the same pathogenic bacteria. It appeared (Figure 3b,d,f) that the three bacteria tested did not attach to the surfaces obtained from the hydrogenated oligomers in comparison to the acrylate and the epoxidized ones, independently from the hydrogenation ratio, while the number of colonies remaining in suspension was not substantially different from the control one. The surfaces obtained from the epoxy oligomers with 30% of epoxy groups had no different behavior in comparison to the control polystyrene; the higher percentage of epoxidation provoked an improvement of activity in comparison to the simple acrylates only for SE.

In conclusion, the hydrogenation or epoxidation ratio made a difference in the surface properties that affected the bacteria behavior. The surfaces from partially hydrogenated oligomers were the best in inhibiting bacteria attachment without any biocide action, as the number of colonies in suspension was similar to the control. The hydrogenated surfaces were the most hydrophobic and less polar of the three surfaces tested; the epoxidized surfaces were the most polar and hydrophilic, and also the least active (except for SA). As for the acrylate surfaces **(1)**, they were in between the two others, regarding the AF action and polarity. For instance, contact angle and surface energies had been previously measured and gave 7% polarity for acrylate **(1**), as compared to 25% polarity for 30% epoxidized acrylate oligomers [20,21].

Some samples presented values bigger than the control (see also Figure 2). Apart from possible pipetting errors (especially when the number of cells is twice that of the control), no satisfactory explanation can be provided. It is worth noting that for this series of experiments, overall, the total number of bacteria adhered to the surface accounted for *ca.* 3% (0.031 ± 0.010, *n* = 20) of the total number of bacteria in suspension in the control (polystyrene) wells, a mean value twice higher than the percent of cells counted at the surface of the different polymers (*ca.* 0.015 ± 0.007). Although for these experimental conditions (set-up, species, duration), most of the bacteria stayed in suspension, with only a few percent of cells able to adhere to the surfaces, this general trend confirms that, whatever the strains and the polymers considered, the NR-based coatings do present an AF activity.

### 2.3. Comparison of the Action of a Tertiary Amine versus a Quaternary Ammonium Group

In previous work [16,18,20,22], in order to prevent biofouling, the strategy of immobilizing a biocide on the coating surface was adopted. The quaternary ammonium group is widely known to have biocidal activity [23] therefore it was considered for covalently functionalizing the natural rubber derived oligomers. Several structures were designed and used in the synthesis of polyurethane films or in the synthesis of coatings by radical or cationic photopolymerization. Different biological contact tests showed that the QA group retained its biocide ability even when incorporated in the polymer matrix. This effect was confirmed and discussed in Section 2.1 in the present work, using a different methodology to perform the attachment studies, more quantitative than in the former work. However, as the synthesis of the ammonium oligomers requires a certain number of steps, the question arose if the tertiary amino group precursor of the quaternary ammonium would have a similar activity, whilst avoiding the last quaternization step. Moreover the legislation [24] is aiming at the interdiction of the use of QA moieties, because of the toxic effect on the environment in case of ageing of the material and of the consequent release. Therefore we have anticipated shifting towards other biocide groups, even if no release of ammonium salts in the medium occurs with our polymers.

**Figure 4:**
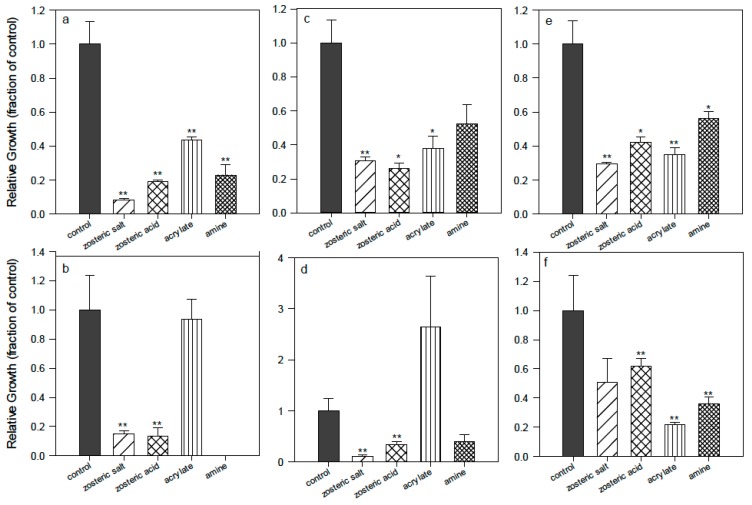
Comparison of basic activity of surfaces from acrylate **(1)** oligomer, from amine **(4)** oligomer, from acrylate mixed with zosteric acid powder (ZA) or with zosteric sodium salt powder (ZAS). *Pseudomonas aeruginosa* (**left**), *Staphylococcus aureus* (**middle**), *Staphylococcus epidermidis* (**right**). (**a**), (**c**), (**e**) = bacteria suspended in the supernatant media; (**b**), (**d**), (**f**) = bacteria attached to the control (polystyrene) or polymer surface. *, difference in values significant at *p* ≤ 0.05, ** difference in values significant at *p* ≤ 0.01.

The amine oligomer **(4)** (Figure 1) is the precursor of the ammonium oligomer **(5)**. It was tested in the same conditions and it displayed a different influence depending on the bacterium (Figure 4). For PA the effect was the most relevant as almost no bacterial cells adhered to the polymer surface and few remained in suspension. For SE there was a pronounced effect in comparison to the control polystyrene but it was slightly less effective than the acrylate alone; for SA there is a small effect in comparison to control and acrylate. If the activity of the amine oligomer **(4)** is compared to the QA oligomer **(5)** (in Figure 2) it is clear that the ammonium has a much stronger effect; however it could be envisioned in future work to obtain surfaces from the mixture of different percentages of oligomers, introducing the amine oligomer **(4)** together with another one containing the biocidal group. An optimal formulation could be found, taking into account the benefit of reducing the number of synthetic steps in view of scale-up and real applications.

### 2.4. Dynamic Tests and Modification of Surface Thickness by Spin Coating

**Figure 5:**
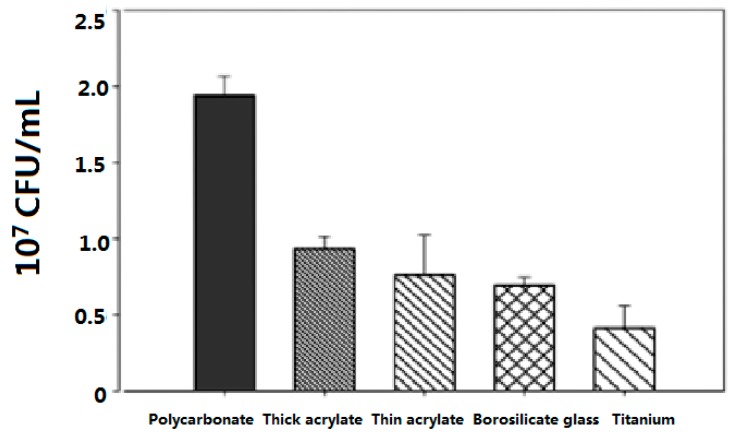
Dynamic tests in flow cells: comparison of *Pseudomonas aeruginosa* attachment on acrylate **(1)** surfaces made by spin coating on borosilicate glass coupons (thin = 45 nm polymer thickness, thick = 0.1 mm) with other non coated materials (polycarbonate, bare glass coupon and titanium).

Different methods of performing the biological assays were used during the project. For the initial screening between the surfaces, the method of depositing polymer disks on an agar plate, covering with bacteria and observing the formation of an inhibition halo was adopted. In this quick and simple way, it was possible to see if bacteria had grown on the polymer surface and if there had been any leaching. However this technique is not quantitative and it was decided that more information could be obtained by counting the colonies formed on the surface after incubation and separation of the supernatant. Both techniques involved testing the polymers in static conditions, which is not characteristic of realistic environments. Therefore supplementary biological tests were performed in dynamic conditions. The available equipment was a flow cell with two lanes (BioSurface Technologies, Corp. (Bozeman, MT, USA), BST FC 271), which could hold two coupons in each lane, one was upstream from the other, not touching each other but with a space between. These coupons were round borosilicate disks (RD 128-GL), of 12 mm diameter and 5 mm height and they could fit into the flow cell only if the polymer film deposited on their surface was very thin. The form of the coupons as well as the necessity of having coatings with a low thickness required the deposit of the pre-polymerization mixture by spin coating. The acrylate **(1)** oligomers and the photoinitiator were dissolved in tetrahydrofuran (THF), the solutions were spread by spin coating on the glass disks, and they were photopolymerized after solvent evaporation. Parameters such as oligomer concentration, spinning time and rate were optimized to have thicknesses in the range 2–100 nm. For the dynamic test, the polymer called “thin acrylate” (Figure 5) was obtained with an oligomer concentration of 20 mg/mL, and a rate of 1000 rpm, while the “thick acrylate” was obtained by spreading the bulk pre-polymerization mixture at 3000 rpm for 30 s. The spin coated thin films were studied by X reflectivity (Figure S3, Table S1), prepared from two different oligomer chain lengths (1200 g·mol^−1^ for films A,B,C,D,F and 1700 g^−1^·mol for films R,X,Y,Z,W). The electron density of spin-coated films was found to be around 0.38 electrons/Å^3^, in good agreement with the calculated one from the polymer formula and volume density. Spin-coated films divided into two classes: Thin films with 40–46 nm thickness, with a roughness around 5 nm and the presence of surface inhomogeneities in the form of surface domains (Figure S3a); and ultra thin films (2–3 nm, Figure S3b), more homogeneous laterally and with a low roughness comparable with the glass one. Atomic Force Microscopy (AFM) images were taken of the coupons bare glass surface and of a thick NR coating and they showed island domains (nanodroplets in pseudo-partial wetting), with thicknesses below 50 nm (Figure S4). A roughness of 1.6 nm was found for the uncoated coupon. The presence of polymer nanodroplets and reorganizations of the film during the UV-treatment is not excluded and we envisage for future development of thin films coatings to covalently graft the oligomers to the surface of the substrates during the thin film deposition, to reduce the surface inhomogeneities and prevent adhesion problems.

The coupons covered by the polymer films were placed into the first lane of the flow cell and different control materials in the second one. A suspension of PA in 10% tryptic soy broth medium was introduced and left in static conditions, at 37 °C, for 1 h in order to allow the PA time to attach, and then the flow was started and left for 24 h, with a 3 mL/h flow rate. Polystyrene could not be introduced in the system as control as in the static tests, and it was replaced by polycarbonate, on which was found the highest number of attached bacteria. In these conditions the NR-derived surfaces retained an inferior number of bacteria in comparison to the control but this number was similar to the bare glass. The fact that no significant difference was observed between a thin coating of 45 nm thickness and a thicker one of 100 μm is interesting because the mechanical properties, morphology and roughness of the surfaces are quite different when the thickness changes. This indicates that for the simple acrylate surface, in a continuous flow, the roughness is not a drastic parameter that would influence the interaction with the bacteria cell wall. The number of colony forming units (CFU) on the NR coatings was also not very different from the one on titanium, which was the most effective in the attachment inhibition. The NR coatings did not contain any biocide group so no effect was expected, however, as in the case of static tests, a small activity was observed in comparison to another commercial polymer as polycarbonate, which is used in medical applications as baby incubators, dental or surgical instruments, sterilization boxes and endoscope disinfectant systems. This preliminary dynamic study indicated also that the NR surfaces have a similar behavior to titanium, which is used in medical devices [25] as implantable chambers to treat cancer, dental implants, and artificial joints. This suggests interesting perspectives for applications of the NR coatings in the biomedical field as, if bacterial inhibition is reduced by a basic surface containing only the polyisoprene backbone, it has been demonstrated that the antimicrobial activity can be enhanced by binding covalently a biocide moiety. Hence in the follow up of this work, the tests in dynamic conditions will be continued with NR coatings containing ammonium or other new bioactive groups and the results will be related to surface thickness, smoothness, polarity and species of bacterium.

### 2.5. Boosting Antifouling Action by Incorporation of Zosteric Acid or Zosteric Salt

Having assessed the baseline antibacterial activity of simple surfaces from acrylate oligomers **(1)**, the synthesis of films containing the NR oligomers and zosteric acid (ZA) or zosteric acid salt (ZAS) was performed for the first time. ZA, a sulfoxy ester of coumaric acid, is a natural product extracted from the eelgrass *Zostera marina*; it is water soluble, completely biodegradable and it non-toxic towards larval fish. When embedded in silicone and successively released, it was found (White [26]) that in presence of ~2 μM ZA, barnacle larvae did not attach to surfaces and were not killed. It appears that ZA does not inhibit the growth of fouling bacteria, but it blocks attachment on surfaces and it blocks adhesion of a diversity of fungal and algal spores, and quagga mussels as well [27]. The preliminary results presented in this research work concern the approach of mixing the ZA or ZAS powders with the liquid oligomers and the photoinitiator, in order to obtain antifouling coatings by photopolymerization. The synthesis of both the acid and the salt forms has been carried out because they have a different solubility in water. As in this study, the objective was to incorporate the solid powder and to observe if it was released by the hydrophobic polyisoprene matrix; it was judged useful to start from the two forms, as in the other published studies it was not specified which compound was used.

The synthesis of ZA has been realized to overcome the long procedure of its extraction and purification from the marine plants. The one involving the reaction of *para*-coumaric acid with a complex formed by sulfur trioxide and pyridine in dimethylformamide (DMF) was chosen [28] (Scheme S3). The product was obtained as a white powder, constituted by the disodium salt of the zosteric acid, with 98% yield, and it structure has been confirmed by ^1^H and ^13^C-NMR, FTIR and high resolution mass spectroscopy (Figure S8). The zosteric acid was obtained as a white solid by acidification of a ZAS solution to pH 2 with HCl 2N. The ^1^H-NMR spectrum showed that a mixture of ~95% zosteric acid and ~5% *p*-coumaric acid was obtained (Figure S8). Both the zosteric acid and the salt forms were used in the film formulations.

The idea of incorporating ZA into a polymer and to study the effect of its release was inspired by previous studies [29], in which ZA was incorporated into silicone. The authors measured the minimum amount of ZA that had to be charged and successively released to reduce the attachment of a Lake Erie bacterial consortium and *Pseudomonas putida* by more than 90% in comparison to the untreated silicone. For our series of experiments, the silicone was replaced by the NR-based films formed by acrylate **(1)**, in which the ZA or ZAS were incorporated as powders, and the pathogenic bacteria PA, SE, and SA were used instead of the freshwater ones. The polymer films were obtained on the bottom of the wells of a 24-well cell culture plate and the attachment experiments were carried out in the same static conditions described in Section 2.1. It was found that PA and SA (Figure 4) adhesion was significantly reduced by the presence of ZA and ZAS in comparison to the control and to the acrylate **(1)**, while for SE the effect was more mitigated. These data indicated that ZA and ZAS were partially released from the polymeric substrate because, in absence of any leaching, the same activity of empty acrylate surfaces should have been obtained. Moreover the relative amounts were sufficient to exert a significant effect on the bacteria. No relevant difference was observed between the behavior of the acid and the salt form, meaning that, in buffer medium, both starting powers led to the same protonated/unprotonated structures in equilibrium. Hence, it was concluded that in future studies the disodium salt coming from the first step of the synthesis could be used directly without the need of the acidification step.

The prepolymerization suspensions were prepared considering that 100 mg were introduced in each well as average, and the percentage in weight of ZA or ZAS per well was 5%, which means that ~0.2 μM ZA or ZAS was the maximum amount that could be released. The visual observation of the coatings after immersion in the culture medium, at 37 °C, for 3 h, showed that not all the ZA or ZAS powders were released. Hence the effect of the inhibition has to be attributed to a partial leaching from the hydrophobic polyisoprene matrix, which does not swell significantly in water, in addition to the basic effect of the matrix itself. In a previous work [29], the ZA was dissolved first in water, then in an organic solvent to improve its miscibility with the silicone precursors, as it is generally soluble in organic solvents. In the present work it was preferred to have a dispersion of the solid powder in the NR oligomers rather than a solution because of the difficulty of getting rid of the solvent before the photopolymerization, and the additional problem of verifying that all the solvent had been completely eliminated before the biological assays. These positive results encourage a follow-up of this exploratory work, which will concern the optimization of the amount of ZA or ZAS to load into the polymers and a quantitative study of the leaching as a function of different parameters, such as film thickness. The covalent coupling of ZA and NR oligomers bearing hydroxyl chain ends was also attempted, exploiting the presence of the carboxylic group of ZA to form an ester function with the hydroxyl groups in NR oligomers. Although the first attempts did not give the aimed product, this covalent approach will be pursued in order to be compared with the “leaching” approach and to choose the more long-lasting and efficient coating.

### 2.6. Effect of Acrylate Surface on Marine Bacteria

Bacteria participate in the first irreversible phase of the microfouling process, which usually is involved in the subsequent attachment of bigger organisms, responsible for the macrofouling process [30,31]. In certain cases, the presence of biofilm can facilitate or inhibit the settlement of macroorganisms [32,33]. Therefore the control of bacterial adhesion and biofilm formation is usually considered a prerequisite for the control of biofouling. Furthermore, microfouling development on metallic structures can influence the deterioration of metal by micro-organism activity, thus causing microbially-influenced corrosion [34,35,36].

In an earlier work [18], natural rubber-based coatings demonstrated AF activity against five species of marine bacteria, *Pseudoalteromonas elyakovii*, *Polaribacter irgensii*, *Cobetia marina*, *Shewanella putrefaciens*, and the pathogen *Vibrio aestuarianus*. These species, representative of microfouling agents in estuarine and marine environments, were provided by the ATCC collection. The determination of the AF activity was made using the inhibition halo method. To confirm this AF effect, for the present work, a new series of experiments were run using the colony count method [16], and wild bacterial strains, collected either from an intertidal mudflat biofilm, or associated with the corrosion products formed on carbon steel structures [37]. Among these bacteria, the *Shewanella*, *Alteromonas* and *Vibrio splendidus* strains belong to the *Gammaproteobacteria* class, which may account, in association with *Alphaproteobacteria*, for 75% of commonly cultured bacteria from the marine environment [38]. *Gamma-* and *Alphaproteobacteria* were recognized as the pioneering organisms in marine biofilm formation, especially *Alteromonas, Vibrio*, *Pseudomonas* and *Pseudoalteromonas* [39,40,41]. *Vibrio* strains were also detected with molecular methods as dominant bacteria inside corroding marine biofilms formed on carbon steel surfaces [42], suggesting their involvement in the biocorrosion processes. Similarly, *Bacillus* was a commonly identified genus among culturable bacteria associated with the corrosion product layer during the early stages of marine corrosion of carbon steel. *Bacillus* was also described as the dominant culturable chemoorganotrophic bacteria from planktonic communities of estuarine and marine environments [43] and was largely associated with microfouling on ship hull [44]. The *Flavobacteriia* class, which contains the *Flavobacterium* strain, is also common in marine sediments [45] or in coastal water [46,47].

**Figure 6:**
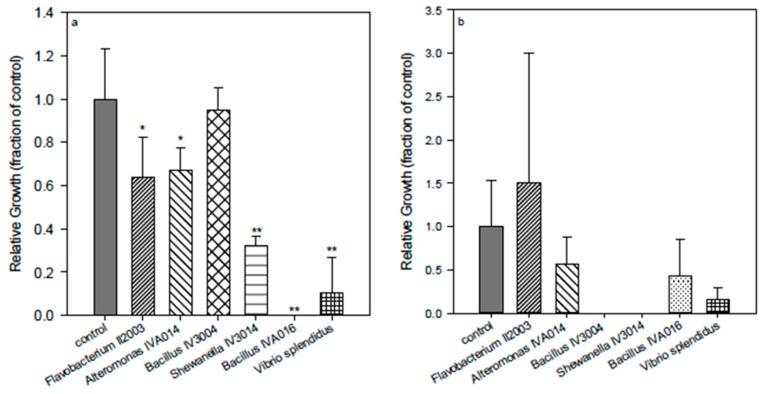
Comparison of basic activity of acrylate **(1)** surfaces using different marine bacteria: *Flavobacterium* II2003, *Alteromonas* IVA014, *Bacillus* IV3004, *Shewanella* IV3014, *Bacillus* IVA016, *Vibrio splendidus*. (**a**) = bacteria suspended in the supernatant media; (**b**) = bacteria attached to the control (polystyrene) or polymer surface. *, difference in values significant at *p* ≤ 0.05, ** difference in values significant at *p* ≤ 0.01.

The antifouling effect of the polyisoprene surfaces was species-dependent (Figure 6), as the adhesion of all species was reduced with the exception of *Flavobacterium* II2003. In addition, *Flavobacterium* was recently identified among the dominant bacteria growing on different commercial biocidal fouling control coatings [48]. *Bacillus* IV3004 (Gram-positive) and *Shewanella* IV3014 (Gram-negative) were very sensitive to acrylate surfaces and presented almost complete inhibition of adhesion and growth on the surfaces. The AF mechanism of action of the surfaces did not seem related to the type of cell wall.

### 2.7. Possible Mechanism of Action for the Anti-Adhesion Effect

The data obtained so far confirmed that the surfaces obtained from NR-derived oligomers containing only the polyisoprene backbone have a weak but noticeable activity *per se*, independently from the oligoisoprene chain length. This inhibition of the bacteria attachment was consistently enhanced if a known biocide group was inserted, such as a quaternary ammonium with at least one long carbon chain on the nitrogen atom. A possible explanation for the basic activity might be found looking at the function, if any, of the latex produced by the *Hevea* rubber trees. The rubber particles suspended in the aqueous latex are a secondary metabolite and the polyisoprene chains are their main constituent. The liquid latex, which is often mistaken for the tree sap, is produced by a system of laticifers, which are chains of contiguous cells connected by anastomoses, and arranged in rings in the secondary phloem. The cells are differentiated from the vascular cambium, and the most important laticifers are in the bark. When the bark is carved, the latex flows out and it rapidly coagulates to generate a solid natural rubber film on the scar. The reason why the tree produces the latex containing the rubber particles is still not clear, however it appears that it has a role of defense from pathogens and herbivorous insects [49]. More generally, latexes can be produced by different plants; they are sticky and viscous liquids, deterrent for insects that try to bite because they are immobilized when they touch the plant surface. Studies have shown that hundreds of proteins are present in the latex, mainly hydrolytic enzymes, and defense or stress-related genes are abundantly expressed during rubber production [50]. Among all these compounds, some latexes contain molecules which are toxic for animals and in some cases they are monoterpenes or diterpenes [51]. Because of the structural resemblance of the polyisoprene backbone with terpenes having a natural antimicrobial activity, we formulated the initial hypothesis [18] that this similarity was the reason for the natural rubber-based surfaces antibacterial effect. With the aim of testing this hypothesis, the sequence of the carbon-carbon double bonds in the NR-based oligomers was modified by hydrogenation or epoxidation, expecting a reduction in the antifouling action of the resulting polymeric films. The results have shown that the suppression of some double bonds can have a small positive influence, but not negative in any case as expected. By partial hydrogenation or epoxidation of the NR oligomers, a modification of the terpenic structure was realized but, at the same time, the polarity of the surface was also changed. It appears that, in our case, the polarity is the key parameter to determine repulsion or attraction, more than the resemblance to natural terpenes. It has been shown [52], however, that it is quite complicated to draw definitive conclusions, because the number of colonies formed on a surface depends not only on the hydrophobicity or the chemical composition, but also on the nutrient level, the age of the microbial culture, the nutrient ability to adsorb on the examined polymeric surface, the ionic strength of the medium, and the static or dynamic conditions in which the attachment experiment is carried out.

## 3. Experimental Section

### 3.1. Materials

NaHCO_3_ technical reagent grade, dry MgSO_4_ laboratory reagent grade, Na_2_S_2_O_3_·5H_2_O, and NaCl general purpose grade were purchased from Fisher (Illkirch-Graffer, France). Periodic acid, DMSO, DMF, Sulfur trioxide pyridine complex (98%), *p*-coumaric acid (98%), LiAlH_4_, triethylamine (≥99.0%), acryloyl chloride (≥97.0%), 2-hydroxy-2-methyl-1-phenyl-propan-1-one (Darocur 1173, 97.0%), were purchased from Sigma-Aldrich (Saint Quentin Fallavier, France) and used without any further purification. Sodium borohydride, Acide 3-Chloroperbenzoïque (70%–75%) were from Acros Organic (Illkirch-Graffer, France) (≥98%). Natural rubber was 10CV60. Tetrahydrofuran (THF), Dichloromethane (CH_2_Cl_2_) were purchased from Fisher and were used as received.

### 3.2. Nuclear Magnetic Resonance and Infrared Spectroscopy

^1^H-NMR spectra were recorded on a Bruker 400 Fourier transform spectrometer at 400 MHz or at 200 MHz. The samples were dissolved in CDCl_3_, D_2_O, using tetramethylsilane (TMS) as an internal standard. The FT-IR spectra were recorded on a Thermo Electron corporation spectrophotomer Nicolet AVATAR 370 DTGS (Thermo Fisher Scientific, Courtaboeuf, France). 

### 3.3. Size Exclusion Chromatography

The number-averaged molecular weight (${\bar{M}}_{n}$) and molecular weight distribution (Đ, dispersity [53]) were measured at 35 °C on a Thermo Finnigan SEC instrument (Thermo Finnigan, Villebon sur Yvette, France, equipped with a SpectraSYSTEM AS1000 autosampler, a SpectraSYSTEM UV2000 and a SpectraSYSTEM RI150 detectors), using a polymer laboratories (PL) gel 5 mm MIXED-D columns, calibrated with a series of standard polystyrenes (580–483 × 10^3^ g·mol^−1^). The polystyrene standardized molecular weights were corrected by the Benoit factor according to the known formula [54]. THF (1.0 mL·min^−1^) was used as eluent and the polymeric solutions injected had a concentration of 5 mg/mL.

### 3.4. Atomic Force Microscopy

AFM images were recorded in the tapping mode with a Nanoscope V from Bruker (Palaiseau, France).

### 3.5. Synthesis of Acrylate Oligomer **(1)**

The synthesis of oligomers **(1)**, **(2)**, **(3)** is reported in Scheme S1.

#### 3.5.1. Synthesis of Carbonyl Oligomers

Solid natural rubber was cut in small pieces (100 g) and then dissolved in 2.5 L of THF in a jacketed reaction flask (5 L) at 30 °C equipped with a mechanical stirrer, for one night, at room temperature. A solution of periodic acid (26.8 g) in THF was added drop-wise into the solution of natural rubber. After this addition, the solution was vigorously stirred for 24 h at 30 °C. At the end of the reaction, the solution was filtered to eliminate the solid residue and evaporated with a rotary evaporator. A sticky product was obtained, which was then dissolved in CH_2_Cl_2_. This solution was washed once with a mixture of a NaHCO_3_ saturated solution/saturated NaCl solution (in volume, 70/30) then once with a mixture of Na_2_S_2_O_3_ solution (20% in weight)/saturated NaCl solution (in volume, 1/1). Then, the organic phase was dried over MgSO_4_ overnight. A yellowish viscous liquid was obtained after filtration and evaporation of CH_2_Cl_2_ (71.8 g; Yield: 72%). *M_n_* = 1700 g·mol^−1^, Đ = 1.90. For the oligomers with *M_n_* 4000 g·mol^−1^, Đ = 2.3, 11.4 g of periodic acid were used for 100 g of NR (yield 70%).

^1^H NMR (CDCl_3_) δ (ppm): 9.77 (s, 1H), 5.10 (s, 1 nH), 2.49 (m, 2H), 2.43 (m, 2H), 2.34 (m, 2H), 2.25 (m, 2H), 2.13 (s, 3H), 2.05 (s, 4 nH), 1.65 (s, 3 nH).

#### 3.5.2. Synthesis of Hydroxyl Oligomers

A suspension of carbonyl oligomers (145 g) and NaBH_4_ (15 g, 65.82 mmol) in THF (2.250 L) was introduced in a jacketed reaction flask (5 L). After stirring overnight at 60 °C, the solution was cooled at room temperature, then hydrolyzed by adding 300 g of ice. This reaction mixture was washed twice with a saturated NaCl solution and dried over MgSO_4_ overnight. The final product was obtained after THF evaporation, as a viscous, yellowish liquid. (130 g; Yield: 89.7%).

^1^H NMR (CDCl_3_) δ (ppm): 5.15 (s, 1 nH), 3.80 (m, 1H), 3.65 (t, 2H), 2.1 (s, 4 nH), 1.7 (s, 3 nH).

#### 3.5.3. Synthesis of Acrylate Oligomers **(1)**

A solution of hydroxyl oligomers 0.08 mol/L (1 equivalent) was prepared in a three-necked flask, in dry dichloromethane, and was cooled at 0 °C, under Argon or nitrogen atmosphere. Triethylamine (2.3 equivalents) and acryloyl chloride (3.0 equivalents) were added dropwise. The reaction mixture was left stirring at RT for 24 h, under nitrogen, then washed with NaOH 1M (for the epoxydated version of the oligomer, a saturated solution of NaHCO_3_ was more effective than NaOH); the organic phase was separated and dried over MgSO_4_, filtered and the solvent was eliminated under reduced pressure. The product was dried under vacuum for 24 h. The ^1^H-NMR spectrum and relative chemical shifts values of acrylate oligomer **(1)** are reported in Figure S5.

### 3.6. Synthesis of Epoxidated Acrylate Oligomer **(2)**

The epoxidation reaction was carried out starting from the hydroxyl oligomers, which were dissolved in DCM and cooled at 0 °C. mCPBA was dissolved in DCM and added to the oligomers. The reaction ran 3 h at RT. The following formula allowed the calculation of the amount of mCPBA to obtain the targeted epoxidation ratio: *m_m_*_CPBA_ = (*m*_PI_/68.8) × *Ţ_e_*/100 × *M_m_*_CPBA_ × (100/70). *m_m_*_CPBA_: grams of metachloroperbenzoic acid, *T_e_*: epoxidation ratio, *M_m_*_CPBA_: molecular weight, 70: purity of metachloroperbenzoic acid, 68.8: isoprene molecular weight (g/mol).

At the end of the reaction, the solution was washed twice with a saturated solution of NaHCO_3_, then the organic phase was dried over MgSO_4_, filtered and the solvent eliminated under reduced pressure. Yield: 53% for 30% epoxidation, 49% for 55% epoxidation.

^1^H NMR (400 MHz, CDCl_3_), δ (ppm): 5.1 (s, 1H, CH=C isoprene) 2.7 (t, 1H CHOC epoxyde) 2.05 (s, 2H CH_2_ isoprene) 1.29 (s, 3H, CH_3_COCH_2_ epoxyde).

*M_n_* = 1300 g/mol, Ð = 1.8 for 30% epoxidation, *M_n_* = 1700 g/mol, Ð = 1.9 for 55% epoxidation.

The synthesis of the final epoxidated acrylate oligomer **(2)** was achieved repeating the step described in Section 3.5.3. The yields for this step were ~90%. The ^1^H-NMR spectrum and relative chemical shifts values of an epoxidated acrylate oligomer **(2)** are reported in Figure S6.

^1^H-NMR (400 MHz, CDCl_3_), δ (ppm): 5.7 (m, 2H, CH_2_ = CH acrylate) 5.1 (s, 1H, CH = C isoprene) 2.7 (m, 1H CHOC epoxide) 2.05 (s, 2H CH_2_ isoprene) 1.29 (s, 3H, CH_3_COCH_2_ epoxide).

### 3.7. Synthesis of Hydrogenated Acrylate Oligomer **(3)**

10 g of hydroxyl oligomers and 1 g of Pd/C catalyst were suspended in ethyl acetate. The suspension was introduced in a catalytic hydrogenation device, and it was mechanically stirred under 3.5 bars in H_2_ atmosphere, at 60 °C, for 21 h. The Pd/C catalyst was then filtered and the solvent eliminated under reduced pressure. The yields for this step were ~90%. 33% hydrogenation was obtained (from NMR calculations). Using 13.1 g of hydroxyl oligomers and 2.04 g of Pd/C catalyst in 100 mL of ethyl acetate, a hydrogenation ratio of 67% was obtained, in the same conditions.

The synthesis of the final hydrogenated acrylate oligomer **(3)** was achieved repeating the step described in Section 3.5.3. The ^1^H-NMR spectrum and relative chemical shifts values of a hydrogenated acrylate oligomer **(3)** are reported in Figure S7.

### 3.8. Synthesis of Amine Oligomer **(4)** and of Ammonium Oligomer **(5)**

The synthesis of oligomers **(4)** and **(5)** is depicted in Scheme S2. The procedure followed is reported in Jellali *et al.* [14]. For oligomer **(4)** and **(5)** *M_n_* = 2900 g/mol, Ð = 1.9.

### 3.9. Synthesis of Zosteric Acid/Salt

Zosteric acid and zosteric acid disodium salt syntheses are depicted in Scheme S3.

The complex pyridine-sulfur trioxide (0.096 mol, 15.3 g, 1.6 equivalents) was added to a solution of *p*-coumaric acid (0.06 mol, 10 g, 1 equivalent) in 18 mL of distilled DMF, under nitrogen atmosphere. The solution was left stirring for 2 h under inert atmosphere, at 50 °C; then it was cooled at RT, and a NaOH solution (20 mL, 30% in weight) was added drop-wise until pH 7. The resulting suspension was filtered, and the aqueous phase was washed three times with 100 mL of dichloromethane. The solution was concentrated under reduced pressure and methanol (250 mL) was added drop-wise causing the precipitation of a white solid. The precipitate was eliminated by filtration and the solvent completely eliminated under reduced pressure to obtain 16.71 g of zosteric acid sodium salt (95% yield). The ^1^H-NMR spectrum and relative chemical shifts values of zosteric acid sodium salt and the IR spectrum are reported in Figure S8.

^1^H-NMR (200 MHz, D_2_O), δ (ppm): 6.36 (d, 1H, *J* = 16.0 Hz), 7.20 (d, 1H, *J* = 8.4 Hz), 7.26 (d, 1H, *J* = 16.0 Hz), 7.52 (d, 1H, *J* = 8.4 Hz).

^13^C NMR (200 MHz, D_2_O), δ (ppm):121.7 (CH), 121.8 (CH), 124.3 (CH), 129.1(CH), 129.2 (CH), 133.1 (C), 139.8 (CH), 151.8 (C), 175.6 (C).

IR, characteristic peaks (cm^−1^): 1645 (C = O); 1245(S = O).

Molecular weight from HRMS was 243.219 g·mol^−1^.

One g of zosteric acid sodium salt was dissolved in water and a solution of HCl 2N was added to pH 2. The zosteric acid was obtained after evaporation of water, 0.83 g were obtained (98% yield).

^1^H-NMR (200 MHz, DMSO), δ (ppm): 6.36 (d, 1H, *J* = 16.0 Hz), 7.20 (d, 1H, *J* = 8.4 Hz), 7.26 (d, 1H, *J* = 16.0 Hz), 7.52 (d, 1H, *J* = 8.4 Hz).

The ^1^H-NMR spectrum and relative chemical shifts of zosteric acid are reported in Figure S8.

### 3.10. Coating Synthesis

For thick films, the pre-polymerization mixture was constituted by the NR oligomers and 5% weight of Darocur 1173 photoinitiator. Fifty to one-hundred mg of it were spread on each bottom of the wells of a 24-well Petri dish and polymerized using a UV-curing equipment (UV Fusion System Corporation, Hanau, Germany), equipped with a hydrogen lamp (power 120 Watt/cm). The belt speed was 1.6 m/min, and the light intensity 2.5 W/cm^2^. Glass slides (2.6 × 2.6 cm) were used as substrates to deposit thin films by spin coating. They were cleaned first with H_2_O, then with acetone, and finally with isopropanol, sonicating in an ultrasound bath, and then they were submitted to plasma treatment. The device that allows the generation of the plasma consisted in photons emitted by a UV lamp that impact molecules contained in the air to activate oxygen into ozone. In this way the links between the surface and the deposited pollutant molecules are broken and the surface is cleaned. The oligomers were dissolved in THF and the photoinitiator was added (5% weight) to have diluted pre-polymerization solutions at different concentrations; these were filtered through a 0.45 μm filter, and deposited on the glass surface by spin coating. Different speeds were applied (from 500 to 2000 rpm) to spread the solution at different concentrations and the photopolymerization was carried out after solvent evaporation irradiating with a UV lamp (wavelength 320–400 nm), equipped with two optic fibers. The light intensity was measured with a UV Power Meters, series C6080, and varied in the range 0.3–11 mW/cm^2^.

### 3.11. Bacterial Strains and Growth Conditions

The Bacterial strains *Staphylococcus epidermidis* ATCC^®^ 35984™ and *Pseudomonas aeruginosa* ATCC^®^ BAA-47™ were purchased from LGC Standards (Molsheim, France), and *Staphylococcus aureus* ATCC^®^6538P™ and *Escherichia coli* ATCC^®^ 8739™ were purchased from Deutsche Sammlung von Mikroorganismen und Zellkulturen GmbH/German Collection of Microorganisms and Cell Cultures GmbH (Braunschweig, Germany). These strains were routinely cultivated in tryptic soy broth (TSB) and tryptic soy agar (TSA) at 37 °C. Marine bacteria used in this work belong to the LIENSs laboratory collection (University of La Rochelle, France). *Flavobacterium* II2003*, Shewanella* IV3014 and *Bacillus* IV3004 strains were collected from an intertidal temperate mudflat biofilm of the French Atlantic coast. *Bacillus* IVA016 and *Alteromonas* IVA014 were isolated from the biofilm associated with the corrosion products formed on carbon steel structures immersed in a French Atlantic harbor [30]. These marine strains were identified by 16S rRNA gene sequencing. They were grown in Marine broth at 22 °C and maintained on Marine Agar (Conda). *Vibrio splendidus* was obtained from the Collection of Pasteur Institute (CIP, 107715). All reagents were purchased from Fisher Scientific (Illkirch, France).

### 3.12. Biological Assays

Attachment assays were performed as previously described [13]. 1 mL of TSB was added to each well to be tested in a 24-well cell culture plate. Two microlitre of OD_600_ = 0.05 overnight culture of the bacteria to be tested were also added to each well. The plates were incubated for 3 h at 37 °C. After the incubation the liquid culture was removed from each plate and diluted and plated for the media sample. The wells were washed three times with sterile water and then fresh TSB was added to the wells and the entire plate was sonicated using Ultrasonic cleaner: 3510-MT (Bransonic, Saint Quentin Fallavier, France) for 5 min. The media was then removed and diluted and plated for the polymer sample.

To enumerate viable bacteria, appropriate dilutions of cultures were made and spirally plated on TSA plates using an Automatic Spiral plater (EasySpiral, Interscience, Saint Nom, France). The plates were incubated for 24 h at 37 °C, and the colonies were then enumerated manually or using an automatic colony counter (Scan500, Interscience, France).

### 3.13. Statistical Analysis

Significance was determined with a 2-tailed *t*-test analysis of variance assuming equal variances. Calculations were done in Excel. Significance was found at *p* ≤ 0.05 and highly significant at *p* ≤ 0.01.

## 4. Conclusions

The objective of this research work was to synthesize coatings from functional telechelic oligomers derived from the fragmentation of the polyisoprene chains of natural rubber and to test their antimicrobial and antifouling activity, leading to a material with two potential different applications. The main features of these new polymeric films are that they are bio-based and non-leaching, because the approach was to link covalently the biocide to the polymeric matrix. After demonstrating that no toxic molecule was released from the coatings in the conditions chosen for the experiments, in this study, the attention was focused in particular on the activity of surfaces without biocide but constituted by the polyisoprene backbone alone. It was found that in static or dynamic conditions they had a weak but significant activity against the biofilm formation of pathogenic bacteria such as *Pseudomonas aeruginosa*, *Staphylococcus epidermidis*, and *Staphylococcus aureus* in comparison to other commercial polymers. The oligomer structure was successively modified by statistical epoxidation or reduction of some carbon-carbon double bonds and it was found that the induced change of the hydrophobicity/hydrophilicity of the surface could decrease attachment depending on the type of bacterium. The influence of the oligomer chain length was studied and it was found that it did not have any effect in the case of the simple oligoisoprene but it was an important parameter for the oligomers containing the biocidal group at the chain end, as the longest chain was the most effective. The surfaces without biocide were also tested with six marine bacterial strains. They also demonstrated a relevant ability in reducing the number of attached colonies, while not permanently damaging the cells, which were able to keep on growing in the culture medium after having been in contact with the polymers and detached. It was confirmed that a method to increase the basic polyisoprene activity was to covalently graft an ammonium group to each oligomer, but different formulations could be envisaged inserting an amount of oligomers bearing the amine precursor of the ammonium, which had a weaker effect in comparison to the latter but required less synthesis steps. Finally, preliminary tests were made, incorporating zosteric acid and zosteric acid salt in the polyisoprene matrix, and it was observed that they were active against the pathogenic bacteria tested.

## Supplementary Materials

Supplementary materials can be found at http://www.mdpi.com/1422-0067/16/3/4392/s1.
