# Supplementary material for: Assessing the Antimicrobial Activity of Polyisoprene Based Surfaces
Source: Int J Mol Sci. 2015 Feb 19;16(3):4392–415. doi: 10.3390/ijms16034392 (PMC4394427; doi:10.3390/ijms16034392)

Supporting information

Figure S1. Thermal gravimetric analysis of a film made from acrylate oligomers **(1)**.

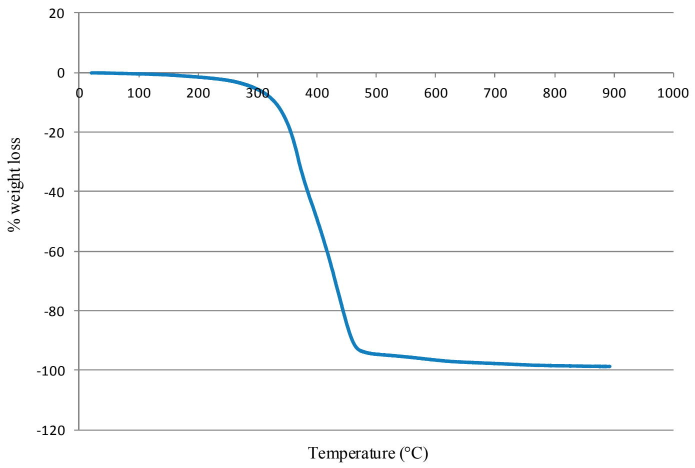

Figure S2. Non-leaching experiment: relative growth of two bacteria, *Staphylococcus aureus* and *Escherichia coli*, in a culture medium that had been in contact for 3 h or 24 h with acrylate **(1)** based coatings. *, difference in values significant at $p \leq 0.05$.

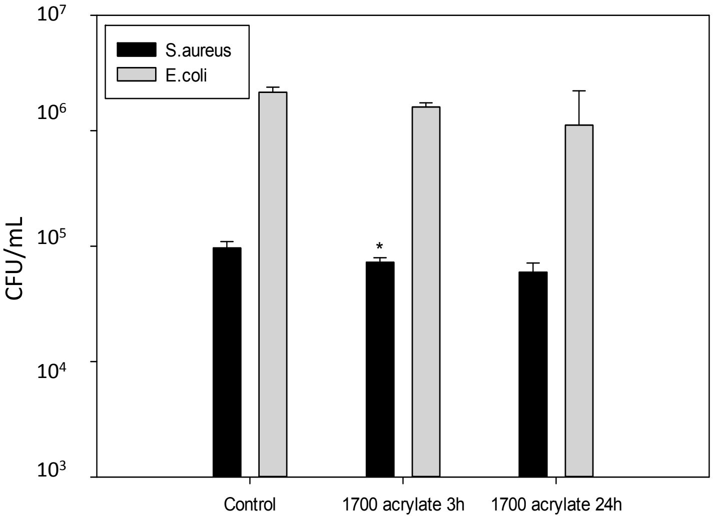

Figure S3. X reflectivity curves of thin acrylate **(1)** spin coated films: (**a**) inhomogeneous films (containing island-like domains) or thick rough films; (**b**) well defined thickness films. Relative data are presented in Table S1.

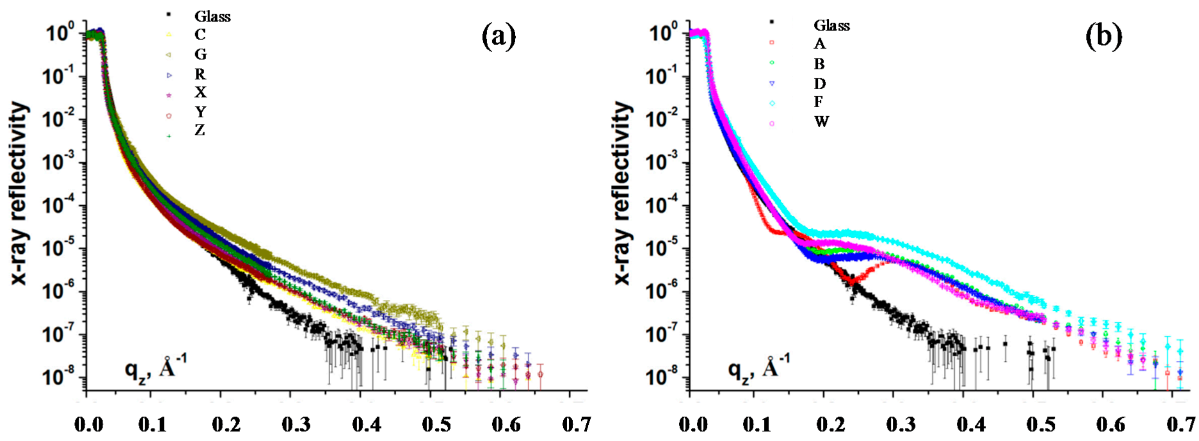

Figure S4. AFM images of: (**a**) bare borosilicate glass coupon surface; (**b**) acrylate **(1)** spin coated film on borosilicate glass coupon.

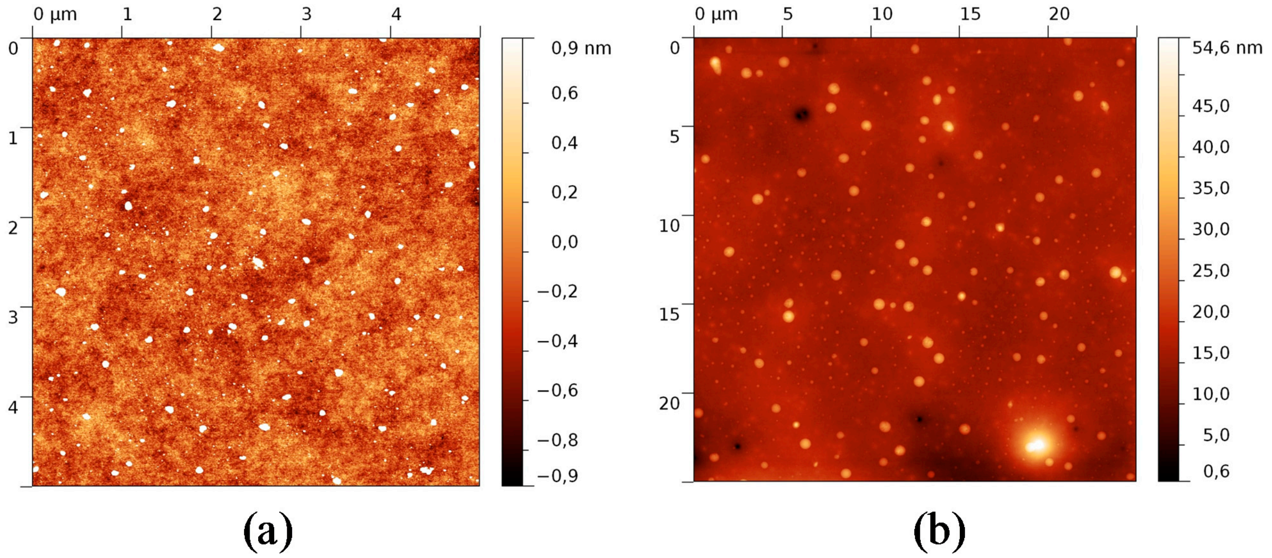

Figure S5. ^{1}H-NMR spectrum and chemical shifts of acrylate oligomer **(1)**.

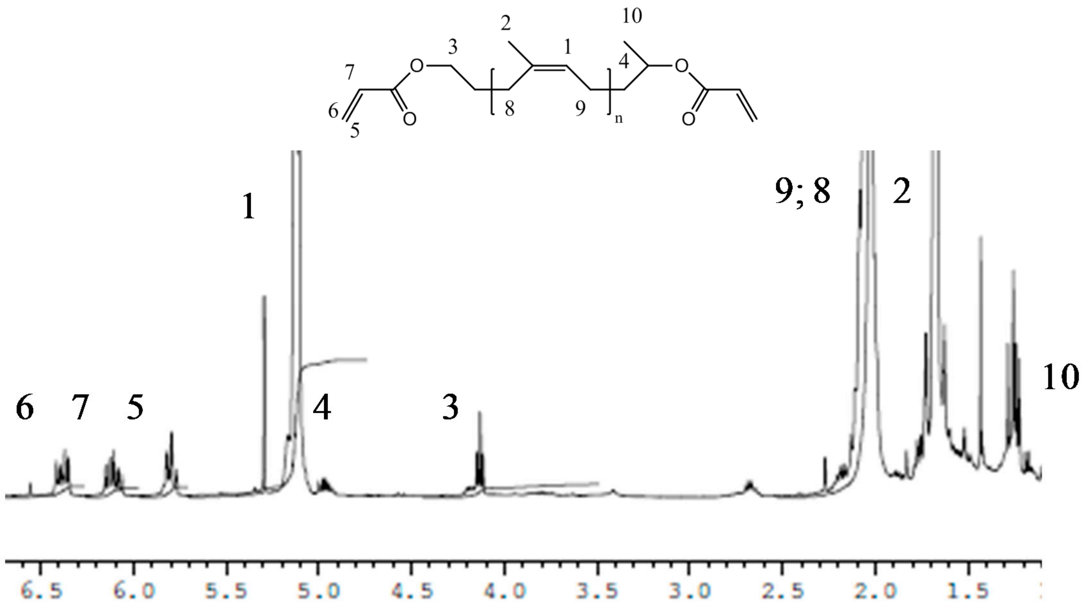

Figure S6. ^{1}H-NMR spectrum and chemical shifts of epoxydated acrylate oligomer **(2)**.

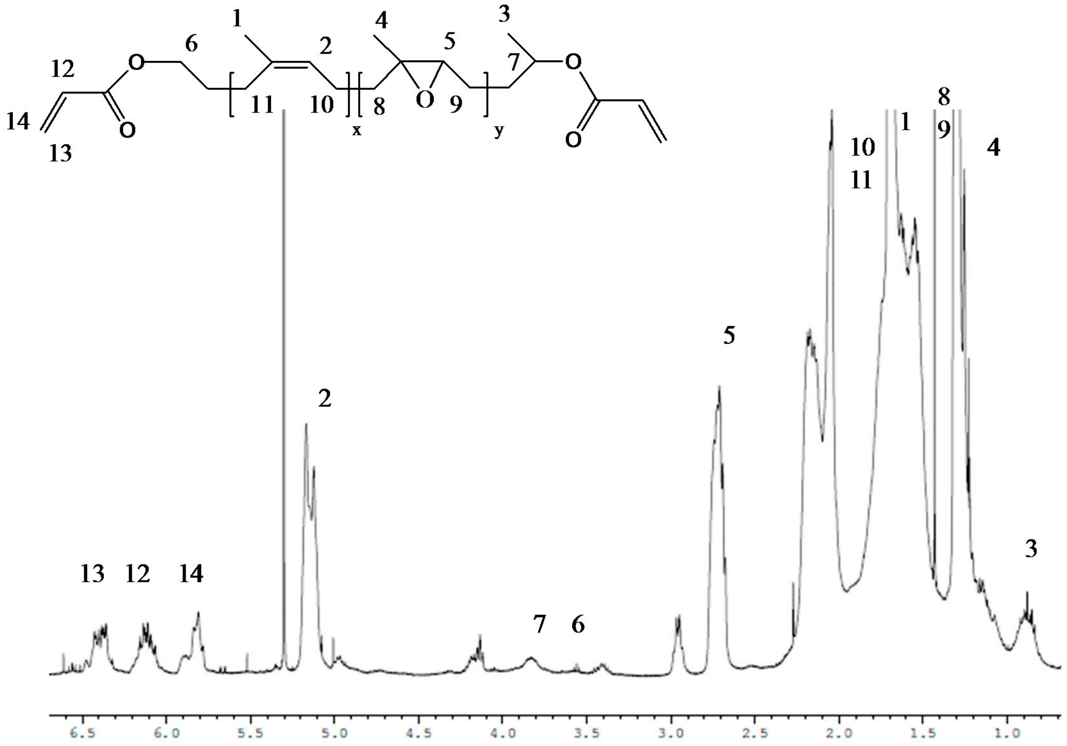

Figure S7. ^{1}H-NMR spectrum and chemical shifts of hydrogenated acrylate oligomer **(3)**.

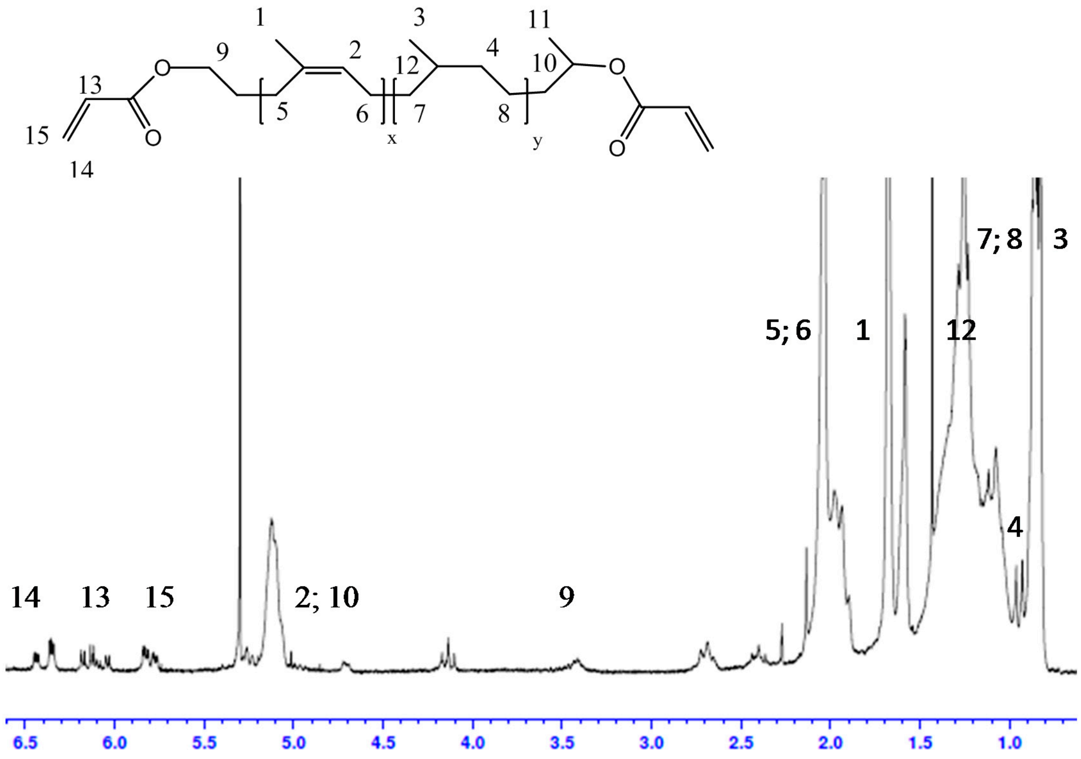

Figure S8. (**a**) ^{1}H-NMR spectrum of zosteric acid disodium salt (200 MHz, D_2O); (**b**) IR spectrum of zosteric acid sodium salt; and (**c**) (200 MHz, DMSO) ^{1}H-NMR spectrum of zosteric acid.

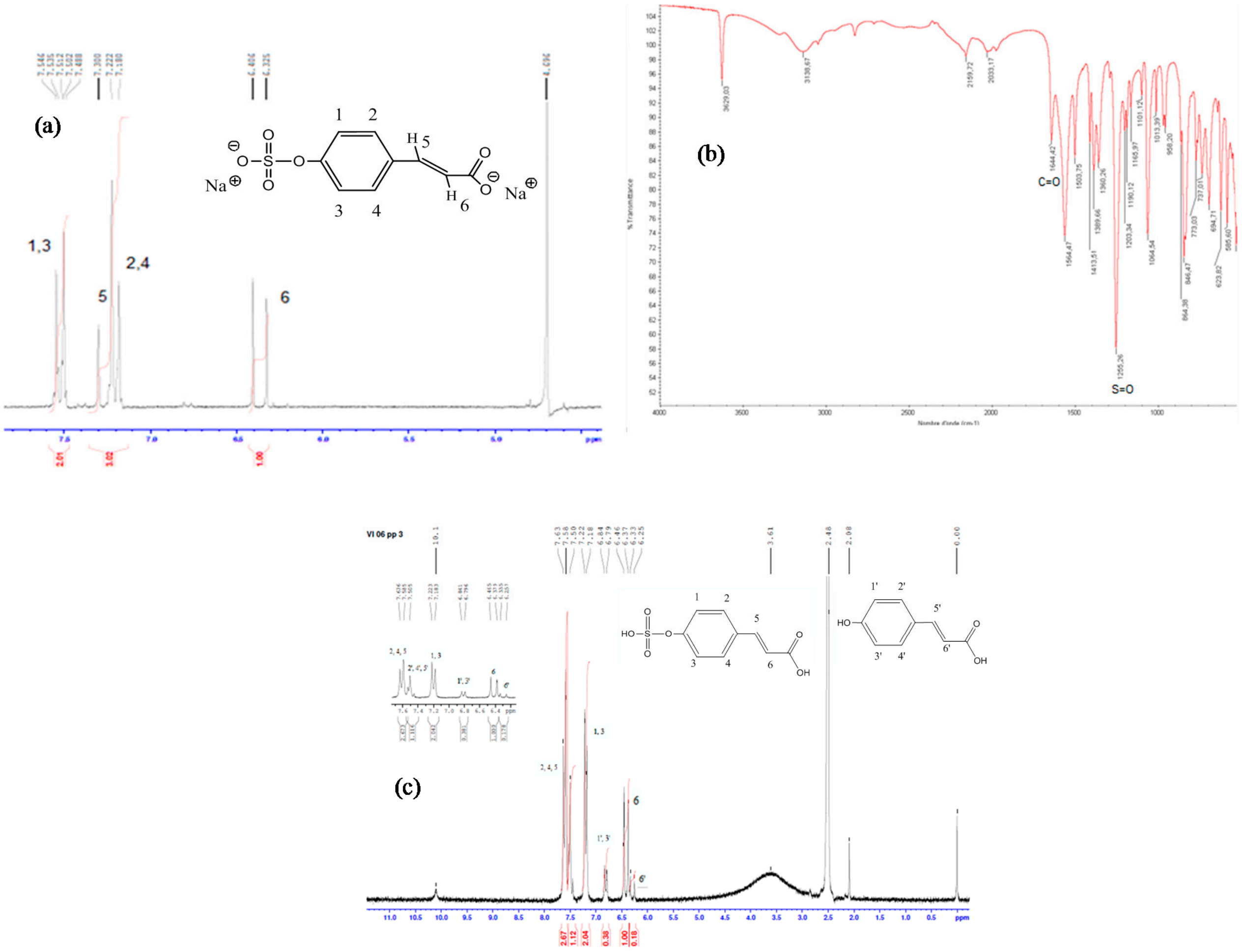

Table S1. Parameters relative to X-reflectivity curves reported in Figure S3.

Thin Samples	Ultrathin Samples	Thickness (Å)	Roughness (Å)
-	A	17	3.5
-	B	18	3
-	C	30	30 (dewetting)
-	D	18	3
R	-	463	49
W	-	418	45
X	-	442	51
Y	-	460	48
Z	-	443	41

Scheme S1. Synthesis of acrylate oligomer **(1)**, of epoxydated acrylate oligomer **(2)** and hydrogenated acrylate oligomer **(3)**.

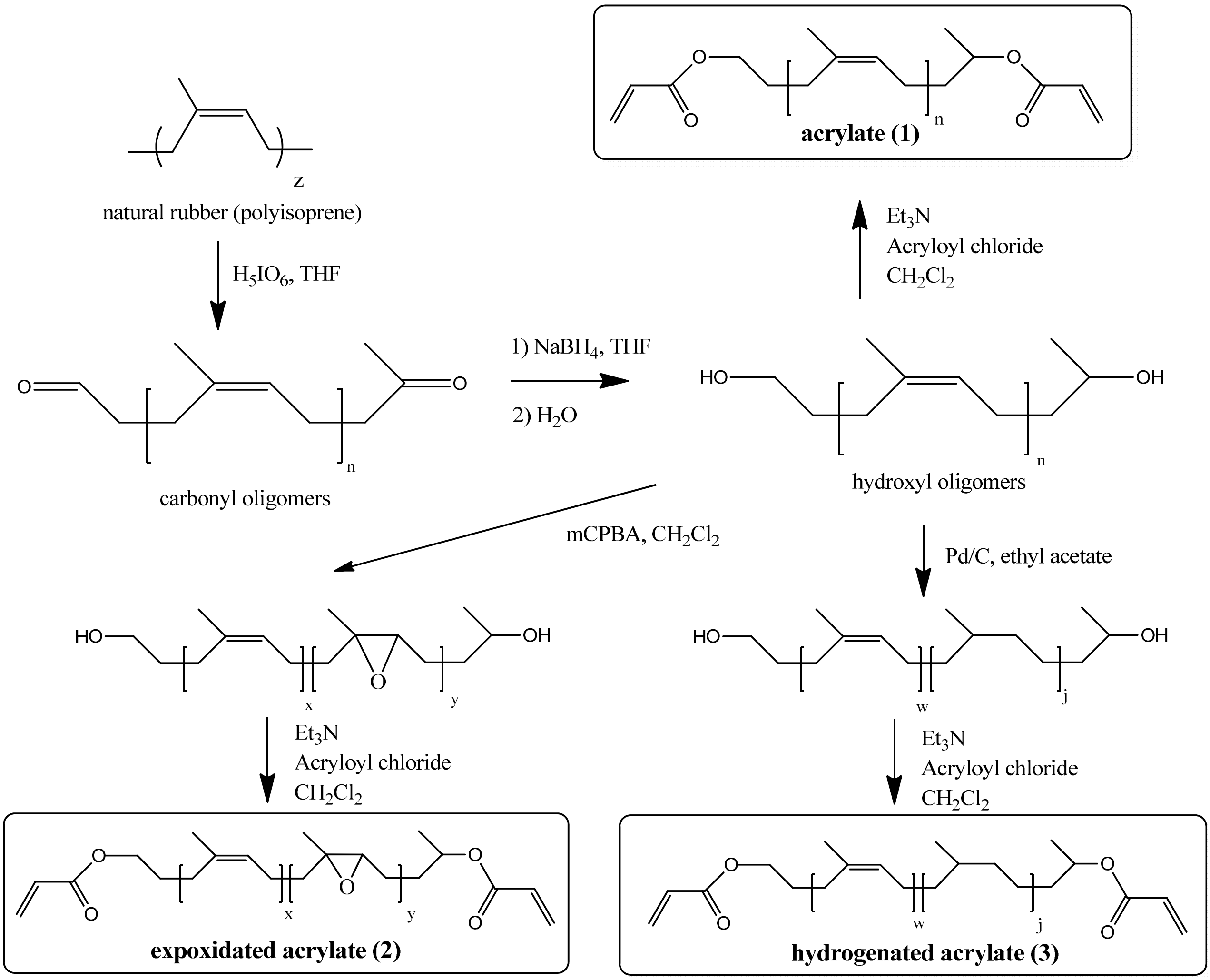

Scheme S2. Synthesis of amine oligomer **(4)** and ammonium oligomer **(5)**.

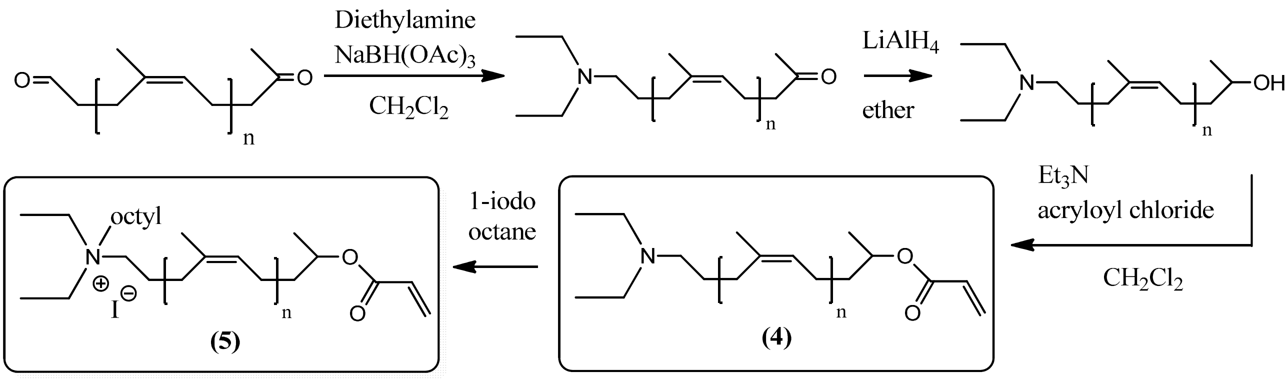

Scheme S3. Zosteric acid and zosteric acid disodium salt (ZAS) synthesis.

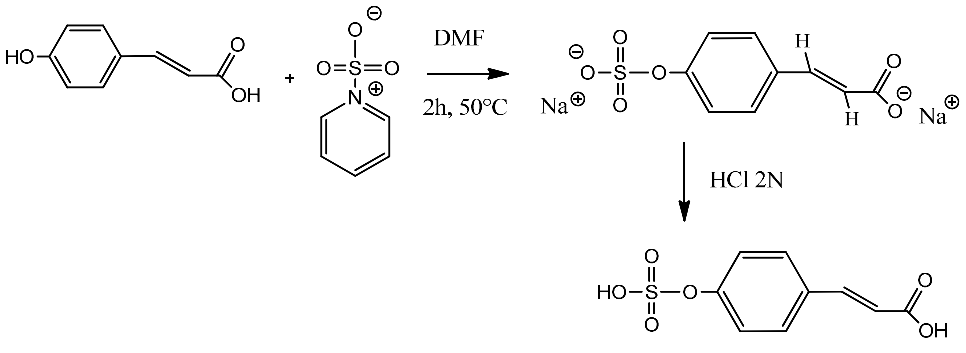

Supplement: Supplementary file 1 [file ijms-16-04392-s001.pdf]
